# Gut Microbiome and Plasma Metabolomic Analysis in Patients with Myelodysplastic Syndrome

**DOI:** 10.1155/2022/1482811

**Published:** 2022-05-09

**Authors:** Huijuan Jiang, Xiaoyu Zhao, Mengtong Zang, Rong Fu, Zonghong Shao, Chunyan Liu

**Affiliations:** Department of Hematology, Tianjin Medical University General Hospital, China

## Abstract

Myelodysplastic syndrome (MDS) is a heterogeneous group of clonal hematopoietic stem cell disorders. Studies have shown the involvement of an abnormal immune system in MDS pathogenesis. The gut microbiota are known to influence host immunity and metabolism, thereby contributing to the development of hematopoietic diseases. In this study, we performed gut microbiome and plasma metabolomic analyses in patients with MDS and healthy controls. We found that patients with MDS had a different gut microbial composition compared to controls. The gut microbiota in MDS patients showed a continuous evolutionary relationship from the phylum to the species level. At the species level, the abundance of *Haemophilus parainfluenzae*, *Streptococcus luteciae*, *Clostridium citroniae*, and *Gemmiger formicilis* increased, while that of *Prevotella copri* decreased in MDS patients compared to controls. Moreover, abundance of bacterial genera correlated with the percentage of lymphocyte subsets in patients with MDS. Metabolomic analysis showed that the concentrations of hypoxanthine and pyroglutamic acid were increased, while that of 3a,7a-dihydroxy-5b-cholestan was decreased in MDS patients compared to controls. In conclusion, gut microbiome and plasma metabolomics are altered in patients with MDS, which may be involved in the immunopathogenesis of the disease.

## 1. Introduction

Myelodysplastic syndrome (MDS) is a heterogeneous group of clonal hematopoietic stem cell disorders, characterized by myelodysplasia, cytopenia, and a high risk of transformation into acute myeloid leukemia (AML). Patients with MDS usually suffer from anemia, hemorrhage, and infection, which are related to poor prognosis. Furthermore, risk stratification, based on clinical and cytogenetic characteristics, divides MDS patients into different risk groups. The number of malignant clones increases in high-risk MDS patients, which could result in AML transformation [1–3]. Many studies have proven that MDS patients have an abnormal immune status. In high-risk MDS patients, there are impaired antitumor immune cells (including dendritic cells (DCs) [4], natural killer (NK) cells [5], and cytotoxic T lymphocytes (CTLs) [6]) and overfunctional immunosuppressive cells (including T regulatory cells (Tregs) [7] and myeloid-derived suppressor cells (MDSCs) [8]) which may facilitate immune evasion of the malignant clones. In low-risk MDS patients, activated inflammatory cells such as T helper 17 (Th17) cells preferentially facilitate ineffective hematopoiesis [9]. Thus, MDS pathogenesis can be attributed to the abnormal immunity; however, its immunopathogenesis still remains unclear.

Recently, the gut microbiota have been shown to play an important role in many diseases. Imbalance of the gut microbiota might disrupt the balance of host immunity or metabolism by changing the permeability of the intestinal mucosa, thereby leading to the development of autoimmune diseases or cancer [10–12]. In hematopoietic disorders, gut dysbacteriosis can affect hematopoiesis via basal inflammatory signaling and is associated with hematologic malignancies [13, 14]. Meanwhile, some researchers have shown that the metabolic signature is altered in hematological diseases. Our previous study demonstrated an altered intestinal microbiome and plasma metabolomics in patients with severe aplastic anemia (SAA), which might trigger an immune imbalance in SAA [15]. However, reports on the profiles of the gut microbiome and plasma metabolomics in MDS are scarce.

In this study, we aimed to investigate the gut microbiome and plasma metabolomics of MDS patients and healthy controls. Further, we intended to assess their relationship with immunocytes in MDS patients to identify potential metabolic markers and novel mechanisms of pathogenesis in MDS.

## 2. Method

### 2.1. Study Subjects

For analysis of the gut microbiota, 15 patients with MDS diagnosed in the Hematology Department of Tianjin Medical University were enrolled from January 2018 to June 2018, including 6 men and 9 women (median age, 65 years; range, 18–69 years). All MDS patients were diagnosed according to World Health Organization (WHO) criteria [16]. They were divided into high-risk (HR, *n* = 7) and low-risk (LR, *n* = 8) groups based on their Revised International Prognostic Scoring System (IPSS-R) scores [17]. The control group included 14 healthy adults, including 3 men and 11 women (median age, 43.5 years; range, 26–63 years) (Supplementary Table 1).

For plasma metabolomic analysis, 10 patients with MDS diagnosed in the Hematology Department of Tianjin Medical University were enrolled from January 2018 to June 2018, including 5 men and 5 women (median age, 59 years; range, 26–71 years). MDS patients were divided into high-risk (HR, *n* = 5) and low-risk (LR, *n* = 5) groups based on their IPSS-R scores. The control group included 10 healthy adults, including 4 men and 6 women (median age, 30 years; range, 25–38 years) (Supplementary Table 2).

This study was approved by the ethics committee of Tianjin Medical University. Written informed consent was obtained from all patients or their family in accordance with the Declaration of Helsinki.

### 2.2. Gut Microbial Analysis

#### 2.2.1. Sample Collection

Participants who did not receive an antibiotic treatment 3 months prior to the date of enrollment were included in this study. Stool samples were collected from each participant within 2 h of consumption of the standard diet. The middle and rear sides of the stool samples were collected using sterile cotton swabs to avoid contamination. Further, 0.5 mL feces of from each sample was collected into a 1.5 mL sterile Eppendorf tube. Two aliquots were collected from each participant and stored at –80°C.

#### 2.2.2. DNA Extraction, 16S rDNA Segment Amplification, and Library Preparation

Microbial genomic DNA from stored stool specimens was extracted using the MagPure Stool DNA KF kit B (Magen, Guangzhou, China) as per the manufacturer's instructions. DNA concentration was quantified using the Qubit®dsDNA BR assay kit (Invitrogen, Carlsbad, CA, USA). The quality of the DNA was assessed by gel electrophoresis (1% agarose) at 150 V for 40 min.

The primer sequences used for the polymerase chain reaction (PCR) were 341F (5′-ACTCCTACGGGAGGCAGCAG-3′) and 806R (5′-GGACTACHVGGGTWTCTAAT-3′). The libraries were sequenced at the Analytical Genomics Core of the Sanford Burnham Prebys Medical Discovery Institute (Lake Nona, FL, USA) and the Beijing Genomics Institute (Beijing, China). The original FASTQ files were processed using the 16S amplicon sequencing pipeline, HiMap (bioRxiv 565572). The HiMap output is an operational strain unit. Read counts were converted into relative abundances. Sample comparisons of each group were performed using log10-transformed relative abundances.

PCR products were purified using the Agencourt AMPure XP magnetic beads (Beckman Coulter, Miami, FL, USA), dissolved in elution buffer, and labelled for library construction. The fragment size and concentration of the constructed library were assessed using an Agilent 2100 Bioanalyzer (Agilent, Santa Clara, CA, USA). Qualified libraries were sequenced on the Illumina HiSeq platform (Illumina, San Diego, CA, USA) to generate 2 × 300 bp reads according to the size of the inserted fragments using the Illumina standard pipeline.

#### 2.2.3. Data Analysis

Following quality control and filtering, the reads were spliced into tags by overlap between the reads using fast length adjustment of short reads (FLASH) software (v1.2.11). The minimum matching length was set at 15 bp. The permissible mismatch rate in the overlapping area was 0.1.

*(1) Operational Taxonomic Unit (OTU) Cluster Analysis*. Taxonomic units (phylum, class, order, family, genus, and species) were identified as OTUs based on unified tags for population genetics analysis. The sequences were clustered into OTUs based on a sequence similarity of >97%. The number of OTUs for each group is presented in a Venn diagram. Partial least squares discrimination analysis (PLS-DA) was used to reflect the differences between the groups.

*(2) Species Composition Analysis*. To obtain species classification, the Ribosomal Database Project (RDP) Classifier, a naive Bayesian algorithm, was used for taxonomic analysis based on the representative sequence of each OTU. The microbial composition of the MDS and control groups was analyzed at the phylum, class, order, family, genus, and species levels, displayed in the form of an abundance histogram. R software (v3.4.1) was used to analyze the relative abundance histograms of the MDS and control groups.

*(3) Diversity Analysis*. Alpha diversity, including species diversity (described by Simpson and Shannon indices) and species richness (described by Chao, ACE, and observed species indices), was used to analyze individual samples. Under conditions of the same species richness, species evenness was proportional to species diversity in the community. A good coverage index was used to describe the library coverage. A higher value indicates a better representation of the actual composition of the sample.

*(4) Differential Species Analysis*. The Wilcoxon rank-sum test and Kruskal-Wallis test were used to determine the abundance of microbial species in the two groups. Statistical significance was defined as *P* < 0.05.

*(5) Association Analysis*. According to the different abundances of microbial species, the Spearman correlation heatmap was drawn using R software. Relationships between the dominant species were represented by color coding. Statistical significance was defined as a correlation coefficient > 0.60 or <–0.60 and a *P* value < 0.05.

### 2.3. Metabolomic Analysis

Fresh peripheral blood samples (5 mL), from MDS patients and controls, collected into EDTA as an anticoagulant, were centrifuged (1600 × *g* for 10 min at 4°C) to separate the plasma. All samples were analyzed using ultrahigh-performance liquid chromatography (UPLC) (Waters, UK). An Acquity UPLC BEH C18 column (Waters, UK) was used for the reverse-phase separation. A high-resolution tandem mass spectrometer (Xevo G2 XS quadrupole-time of flight (Waters, UK)) was used to detect the metabolites eluted from the column, which was operated in both the positive and negative ion modes.

### 2.4. Flow Cytometry

Flow cytometry (FCM) was used to detect clusters of immunocytes in patients with MDS. Fresh whole blood samples collected into EDTA (100 *μ*L) were immunostained with anti-CD3-PerCP, anti-CD8-FITC, anti-CD4-PE, anti-CD56-APC, anti-CD16-FITC, anti-CD19-APC, and mouse isotype controls (BD Biosciences, San Jose, CA, USA) in separate Trucount tubes (BD Biosciences, USA) for 30 min at 4°C in the dark, followed by erythrocyte lysis using 2 mL erythrocyte lytic solution (BD PharMingen, San Diego, CA, USA). After centrifugation at 1300 rpm for 5 min, the supernatant was discarded. Cells were washed with phosphate-buffered saline (PBS) and resuspended in 300 *μ*L PBS, and at least 50,000 cells were acquired on a FACSCalibur flow cytometer (BD Biosciences, USA). Analysis was done using CellQuest software version 3.1 (Becton Dickinson, Franklin Lakes, NJ, USA).

### 2.5. Statistical Analysis

PLS-DA, fold change analysis, and Student's *t*-test were used to analyze the differences in the metabolites between the MDS and control groups. Statistical significance was defined as a fold change ≥ 1.2 or ≤0.8333 and a *P* < 0.05. Spearman analysis was used to analyze the correlation between the abundance of microbial genera and lymphocyte subsets in MDS patients. Statistical significance was set as *P* < 0.05.

## 3. Results

### 3.1. Gut Microbiota Analysis

#### 3.1.1. Composition of Gut Microbiota in MDS and Control Groups

**Figure 1:**
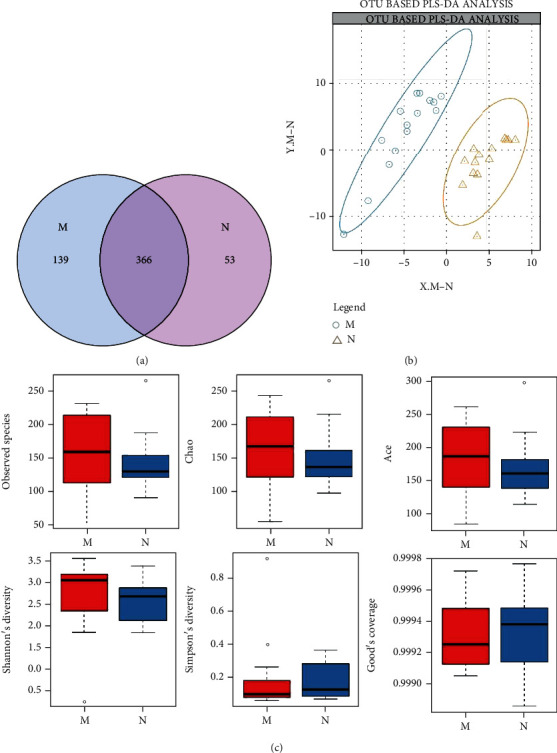
(a) Venn diagram of OTU (M: MDS group, N: normal control group). (b) OTU-based PLS-DA analysis. Orange triangles represent samples (intestinal microbiota) from the normal group; blue circles represent samples from MDS patients. (c) Alpha diversity index boxplot between the MDS group and normal group.

The MDS group contained 505 OTUs, while the control group contained 419 OTUs, as depicted in the Venn diagram. The overlap between the two groups was 366 OTUs (Figure 1(a)). We also found that MDS patients had a different gut microbiota composition compared to healthy controls by PLS-DA (Figure 1(b)).

The mean good coverage values in the MDS and control groups were >99%. This indicated that the sequences obtained from each sample covered almost all bacterial sequences in the library. The MDS group showed higher observed species, Chao, ACE, and Shannon indices and a lower Simpson index as compared to the control group (Figure 1(c)). However, there was no significant difference between the two groups as assessed using the Wilcoxon rank-sum test (Supplementary Table 3).

#### 3.1.2. Microbiota Structures in MDS and Control Groups

**Figure 2:**
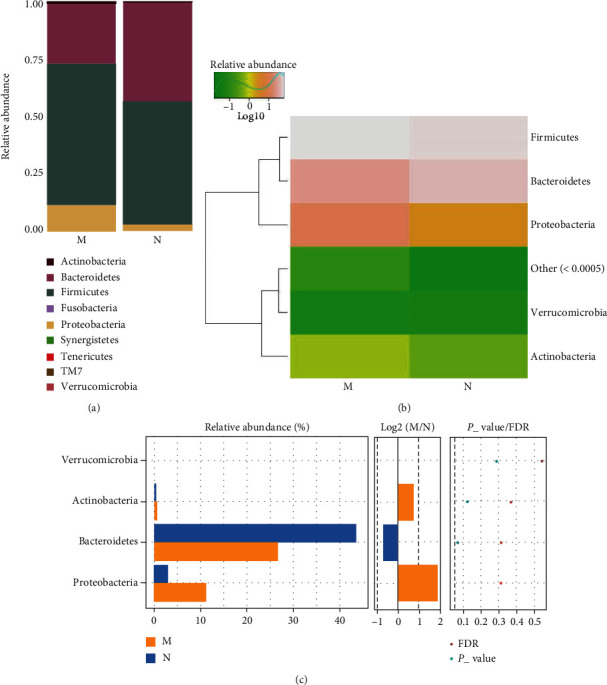
The microbial composition of the MDS group and normal group at the phylum level: (a) bartplot; (b) heatmap; (c) Wilcoxon rank-sum test.

*(1) The Microbial Composition at the Phylum Level*. The MDS group showed an increased and decreased relative abundance of Proteobacteria and Bacteroidetes, respectively, in the gut microbiota at the phylum level compared to the control group (Figures 2(a) and 2(b)). Furthermore, there was a significant difference in the relative abundance of Proteobacteria between the MDS and control groups (*P* < 0.05); however, no significant difference was observed in the relative abundance of Bacteroidetes based on the Wilcoxon test (Figure 2(c)).

**Figure 3:**
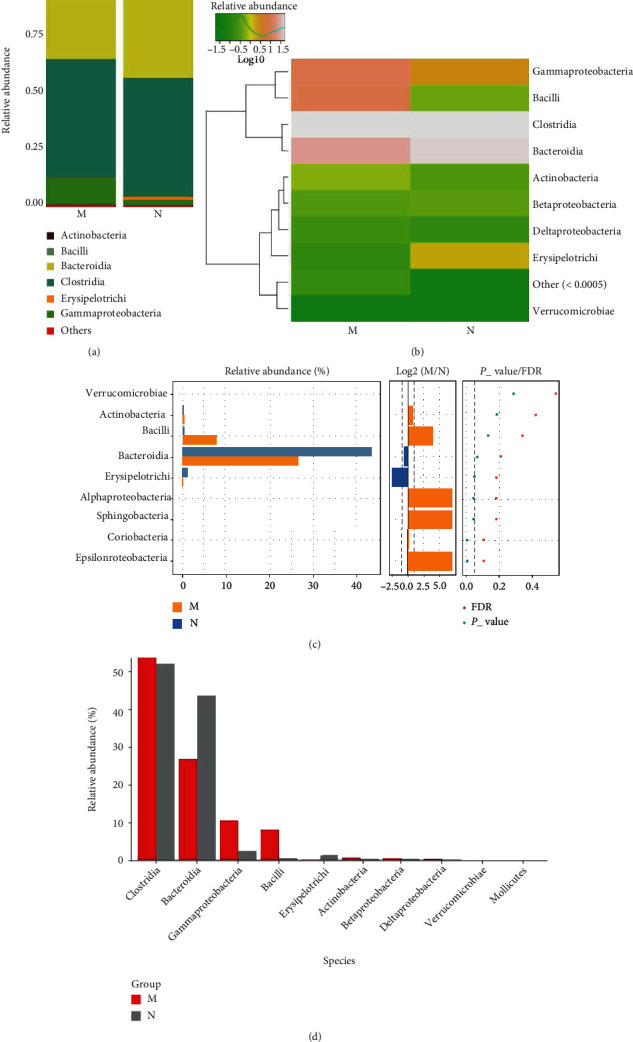
The microbial composition of the MDS group and normal group at the class level: (a) bartplot; (b) heatmap; (c) Wilcoxon rank-sum test; (d) top 10 species.

*(2) The Microbial Composition at the Class Level*. Compared to the control group, the MDS group had an increased relative abundance of Bacilli and Gammaproteobacteria, while that of Bacteroidia and Erysipelotrichi was reduced in the gut microbiota at the class level (Figures 3(a) and 3(b)). However, the difference between the MDS and control groups was not statistically significant as assessed by using the Wilcoxon or Kruskal-Wallis test (Figures 3(c) and 3(d)).

**Figure 4:**
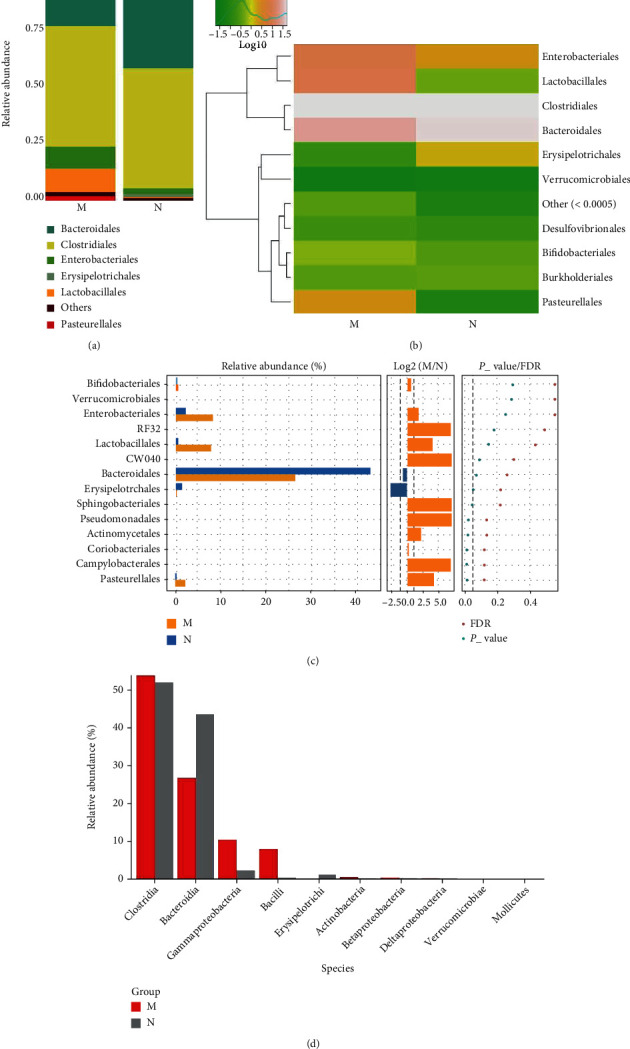
The microbial composition of the MDS group and normal group at the order level: (a) bartplot; (b) heatmap; (c) Wilcoxon rank-sum test; (d) top 10 species.

*(3) The Microbial Composition at the Order Level*. Compared to the control group, the MDS group had an increased relative abundance of Enterobacteriales, Lactobacillales, and Pasteurellales, while that of Bacteroidales and Erysipelotrichales was reduced in the gut microbiota at the order level (Figures 4(a) and 4(b)). The Wilcoxon and Kruskal-Wallis test showed that the relative abundance of Pasteurellales in the MDS patients was significantly higher than that observed in the controls (*P* < 0.05) (Figures 4(c) and 4(d)).

**Figure 5:**
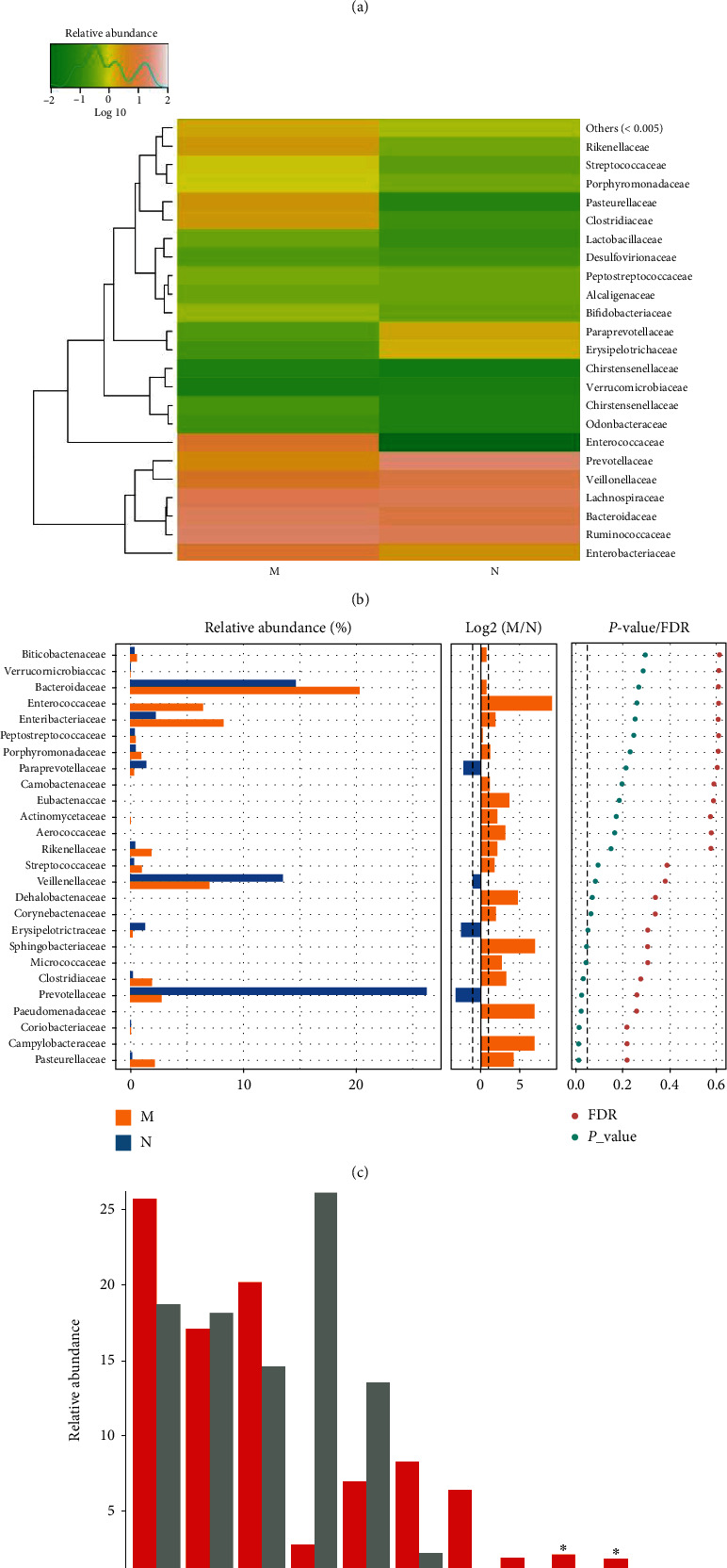
The microbial composition of the MDS group and normal group at the family level: (a) bartplot; (b) heatmap; (c) Wilcoxon rank-sum Test; (d) top 10 species.

*(4) The Microbial Composition at the Family Level*. The MDS group had an increased relative abundance of *Clostridiaceae*, *Enterobacteriaceae*, *Enterococcaceae*, *Pasteurellaceae*, *Rikenellaceae*, and *Streptococcaceae*, while that of *Erysipelotrichaceae*, *Paraprevotellaceae*, *Prevotellaceae*, and *Veillonellaceae* was decreased in the gut microbiota at the family level, as compared to that in the control group (Figures 5(a) and 5(b)). The Wilcoxon test showed that the relative abundance of *Clostridiaceae* and *Pasteurellaceae* in MDS patients was significantly higher than that in the controls, while that of *Prevotellaceae* was significantly lower than that in the controls (*P* < 0.05) (Figure 5(c)). According to the Kruskal-Wallis test, the abundance of *Clostridiaceae* and *Pasteurellaceae*, which were among the top 10 most abundant species, was significantly higher in the MDS group (*P* < 0.05) (Figure 5(d)).

**Figure 6:**
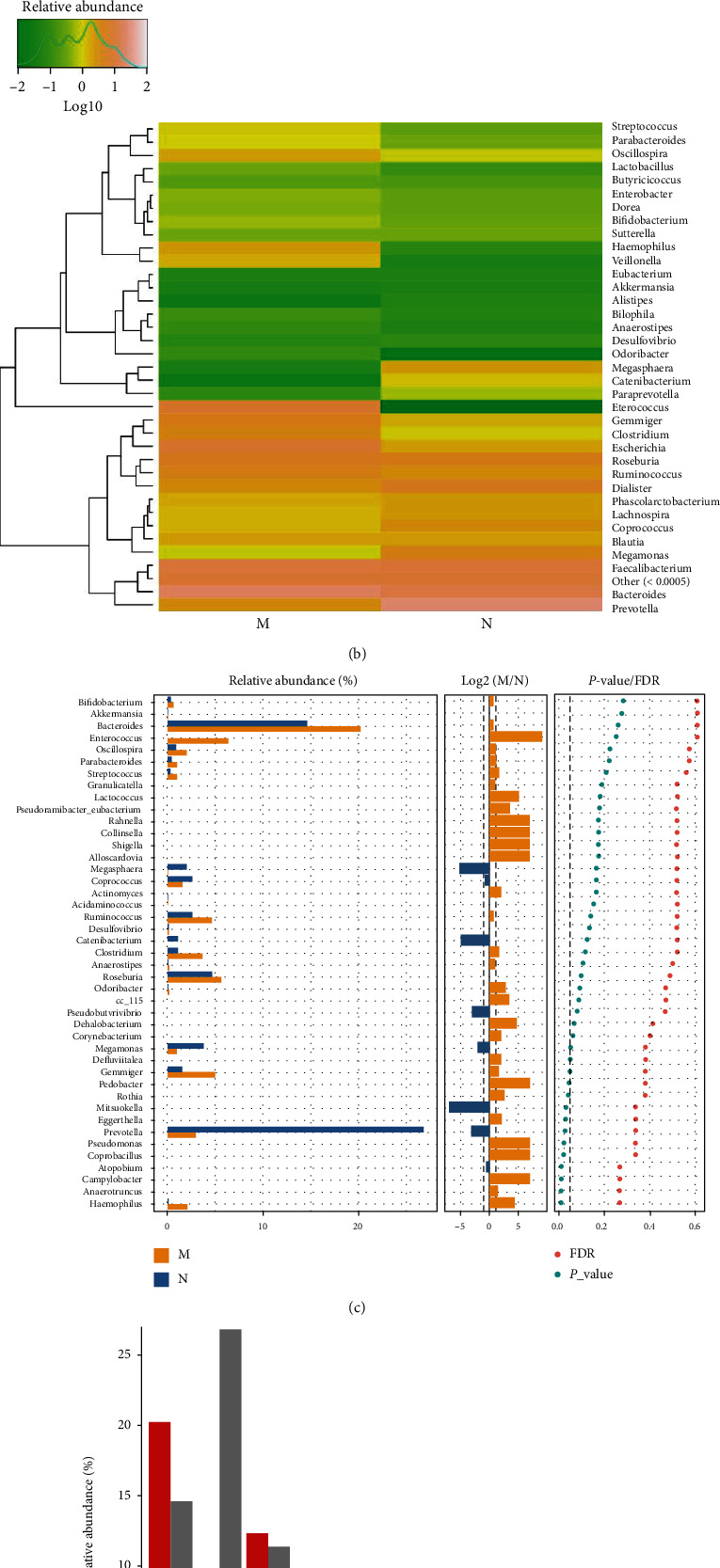
The microbial composition of the MDS group and normal Group at the genus level: (a) bartplot; (b) heatmap; (c) Wilcoxon rank-sum test; (d) top 10 species.

*(5) The Microbial Composition at the Genus Level*. Compared to the control group, the MDS group had an increased relative abundance of *Bacteroides*, *Clostridium*, *Enterococcus*, *Gemmiger*, *Haemophilus*, *Oscillospira*, *Parabacteroides*, *Streptococcus*, and *Veillonella*, while that of *Catenibacterium*, *Megamonas*, *Megasphaera*, and *Prevotella* was decreased in the gut microbiota at the genus level (Figures 6(a) and 6(b)). The Wilcoxon test showed that the relative abundance of *Gemmiger* and *Haemophilus* was higher, whereas that of *Prevotella* was lower in MDS patients than in the controls (*P* < 0.05) (Figure 6(c)). According to the Kruskal-Wallis test, the MDS group had a significantly higher abundance of *Gemmiger* which was among the top 10 most abundant species (*P* < 0.05) (Figure 6(d)).

**Figure 7:**
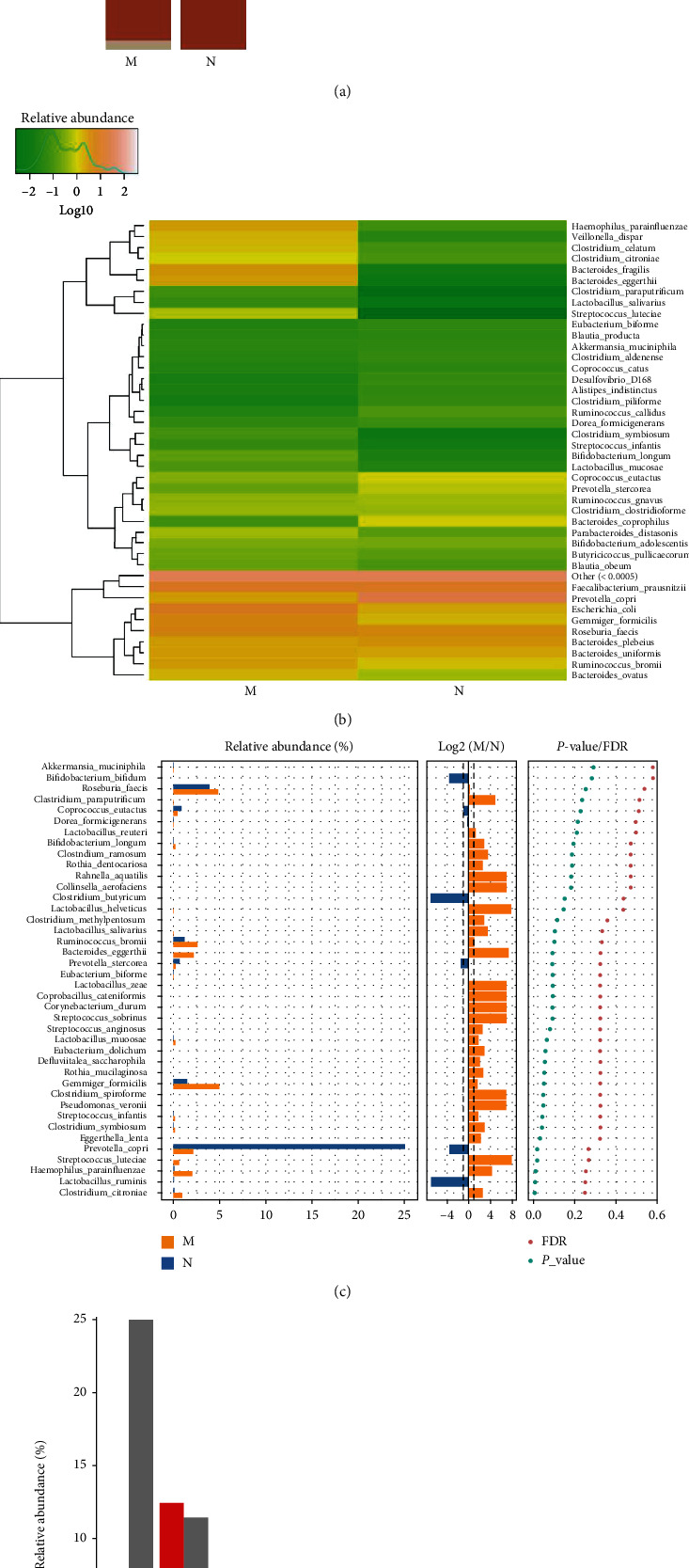
The microbial composition of the MDS group and normal group at the species level: (a) bartplot; (b) heatmap; (c) Wilcoxon rank-sum test; (d) top 10 species.

*(6) The Microbial Composition at the Species Level*. Compared to the control group, the MDS group had an increased relative abundance of *Bacteroides eggerthii*, *Bacteroides fragilis*, *Bacteroides ovatus*, *Clostridium celatum*, *Clostridium citroniae*, *Ruminococcus bromii*, *Escherichia coli*, *Gemmiger formicilis*, *Haemophilus parainfluenzae*, and *Veillonella dispar*, while that of *Coprococcus eutactus*, *Prevotella stercorea*, and *Prevotella copri* was decreased in gut microbiota at the species level (Figures 7(a) and 7(b)). The Wilcoxon test showed that the relative abundance of *G. formicilis*, *H. parainfluenzae*, *Streptococcus luteciae*, and *C. citroniae* was higher in MDS patients than in the controls, whereas that of *P. copri* was lower than that observed in the controls (*P* < 0.05) (Figure 7(c)). According to the Kruskal-Wallis test, the MDS group had a higher abundance of *G. formicilis* and *H. parainfluenzae*, which were among the top 10 most abundant species (*P* < 0.05) (Figure 7(d)).

#### 3.1.3. Correlation Analysis

**Figure 8:**
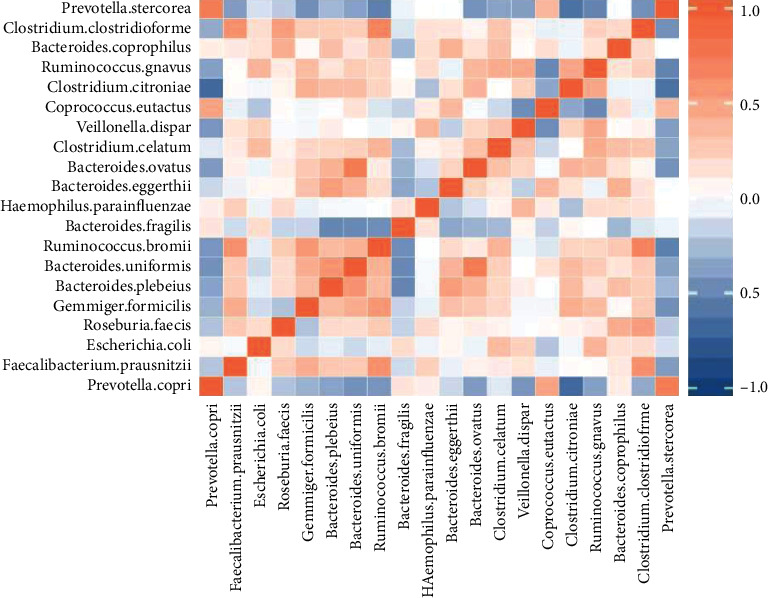
Heatmap of association analysis between the MDS group and normal group.

The correlation between the associated dominant species in the MDS and control groups is depicted in the heatmap (Figure 8). There was a competitive relationship between *P. copri* and *C. citroniae* (*r* = –0.6576, *P* < 0.05).

### 3.2. Plasma Metabolomic Analysis

**Figure 9:**
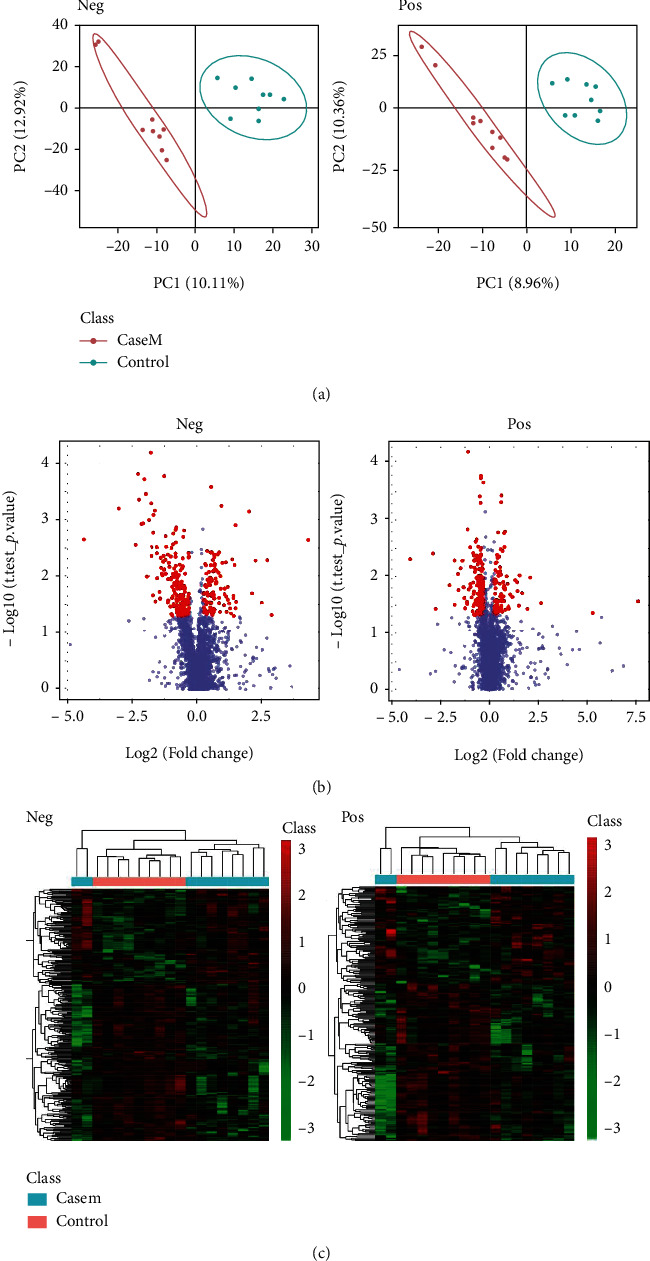
MDS patients had different metabolomics compared to healthy controls in both negative and positive modes: (a) PLS-DA; (b) visual volcanic map; (c) heatmap.

The results are illustrated using PLS-DA (Figure 9(a)), a visual volcanic map (Figure 9(b)), and a heatmap (Figure 9(c)) from the MDS and control groups. A total of three metabolite concentrations differed between the MDS and control groups. The plasma concentrations of hypoxanthine and pyroglutamic acid (PYG) were increased, while those of 3a,7a-dihydroxy-5b-cholestan were reduced in the MDS group (Table 1).

### 3.3. The Correlation between Gut Microbial Composition and Lymphocyte Subsets in MDS Patients

**Figure 10:**
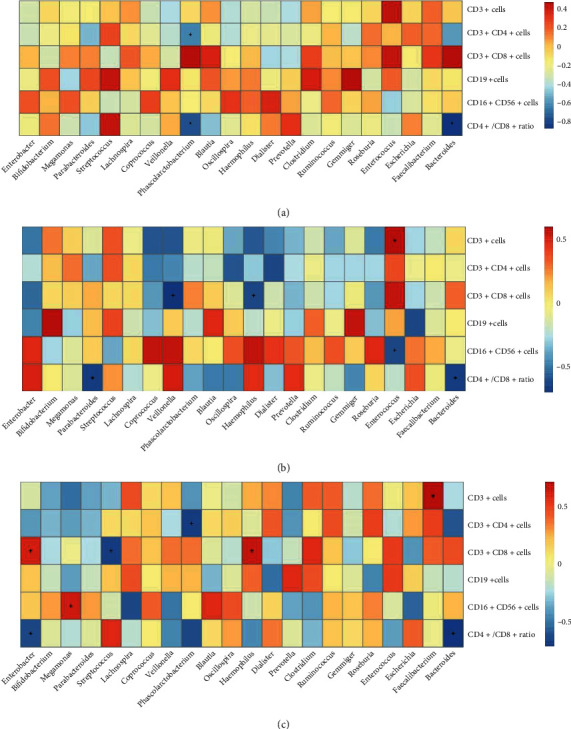
The correlation between the abundance of microbial composition at the genus level and the percentage of immune subsets in MDS patients: (a) total MDS group; (b) HR-MDS group; (c) LR-MDS group (+: *P* < 0.05; ∗: *P* < 0.01).

The percentage of lymphocyte subsets in MDS patients was tested by FCM, including total T cells (CD3^+^), CD4^+^ T cells (CD3^+^CD4^+^), CD8^+^ T cells (CD3^+^CD8^+^), B cells (CD19^+^), and NK cells (CD16^+^CD56^+^). Owing to the large difference of microbes at the species level among the individuals, we analyzed the correlation between the abundance of gut microbiota composition at the genus level and the percentage of lymphocyte subsets in MDS patients. We observed a negative correlation between the percentage of CD4^+^ T cells and the abundance of *Phascolarctobacterium* in the MDS group. The CD4^+^/CD8^+^ cell ratio was negatively correlated with the abundance of *Phascolarctobacterium* and *Bacteroides* in MDS group (Figure 10(a)). We then divided the MDS patients into HR-MDS and LR-MDS groups based on the IPSS-R scores. In the HR-MDS group, the percentage of CD8^+^ T cells was negatively correlated with the abundance of *Veillonella* and *Haemophilus*. Further, the abundance of *Enterococcus* was positively correlated with the percentage of total T cells and negatively correlated with the percentage of NK cells. The CD4^+^/CD8^+^ cell ratio was negatively correlated with the abundance of *Bacteroides* and *Parabacteroides* (Figure 10(b)). In the LR-MDS group, the percentage of total T cells was positively correlated with the abundance of *Faecalibacterium*, while that of CD4^+^ T cells was negatively correlated with the abundance of *Phascolarctobacterium* spp. The percentage of CD8^+^ T cells was positively correlated with the abundance of *Haemophilus* and *Enterobacter* and negatively correlated with *Streptococcus*. There was a positive correlation between the percentage of NK cells and the abundance of *Megamonas*. The CD4^+^/CD8^+^ ratio showed a negative correlation with the abundance of *Bacteroides* and *Enterobacter* (Figure 10(c)).

## 4. Discussion

MDS is a group of malignant clonal hematopoietic stem cell disorders. Several studies have shown that abnormal immunity is involved in the pathogenesis and development of MDS. Impaired CTLs and NK cells [5, 6] and increased MDSCs [8] were observed in MDS patients, leading to the immune evasion of malignant clones and AML transformation. However, the immunopathogenesis of MDS remains unclear.

In recent years, the gut microbiota has received increasing attention owing to its influence on many diseases. Some researchers have found that gut microbiota and metabolomics are related to the pathophysiology of autoimmune diseases including systemic lupus erythematosus and rheumatoid arthritis [18, 19]. The gut microbiota can regulate the immune-inflammatory axis by affecting the activation of immune cells (such as DCs, T cells, and macrophages) and the production of cytokines [20, 21]. In the gut mucosa, T and B cells have position-specific phenotypes that are influenced by the microbiota. Dysbacteriosis may induce an imbalanced immune tolerance, abnormal differentiation of T cells, and altered production of autoantibodies and inflammatory factors in the host [22]. In hematopoietic disorders, gut microbiota also plays an important role in the development and progression of hematological malignancies. In patients with diffuse large B cell lymphoma, fundamental differences in gut microbial diversity and composition were reported [23].

In this study, we found that patients with MDS had a different gut microbial composition compared to normal controls. The gut microbiota of MDS patients showed a continuous evolutionary relationship from the phylum level (Proteobacteria and Bacteroidetes) to the species level (*H. parainfluenzae* and *P. copri*). At the species level, the abundance of *H. parainfluenzae*, *S. luteciae*, *C. citroniae*, and *G. formicilis* increased, while that of *P. copri* decreased in MDS patients compared to controls. Both *H. parainfluenzae* and *S. luteciae* are opportunistic pathogens associated with bacteremia and other infections. The increased abundance of *H. parainfluenzae* and *S. luteciae* in MDS might indicate that MDS patients are at a high risk of developing an infection.

In mouse models, *Clostridium* can increase the Treg population by inducing DC proliferation. In the gut microflora of orally sensitized C3H/HeJ mice, the abundance of *Clostridium* was positively correlated with the levels of CD11C^+^ CD103^+^ DCs and Tregs [24, 25]. *Clostridium* can induce tolerogenic DC-mediated stimulation of Tregs *in vitro* [26], which suggested that it could upregulate Tregs that have an immunosuppressive effect. The proportion of Tregs has been reported to be upregulated in patients with MDS [7]. Thus, we inferred that the upregulation of Tregs in MDS may be attributed to an increased abundance of *Clostridium*, and the mechanism in this regard needs to be addressed in the future.

We also found that the abundance of *G. formicilis* was increased in MDS patients. *Gemmiger* abundance is known to be increased in patients with autoimmune diseases and hepatocellular carcinoma and is involved in the regulation of inflammation [27, 28]. However, the functions of *Gemmiger* in MDS are unclear. Moreover, the abundance of *P. copri* was significantly decreased in MDS patients. In HIV-infected patients, a high abundance of *Prevotella* can activate intestinal myeloid DCs, leading to mucosal and systemic immune activation [29]. Increased *Prevotella* enrichment is associated with augmented Th17 mediating mucosal inflammation [30]. However, the abundance of *Prevotellaceae* is also reported to increase with a decrease in Th17 cells and an increase in Tregs [31]. Despite the variations in different studies, all results suggest that *Prevotella* has regulatory effects on immune cells. The differences observed can be attributed to disease characteristics or microbial interactions. Further studies are necessary to reveal the effect of *Prevotella* on MDS.

Previous studies have demonstrated that an altered gut microbiome can regulate host immunity. Towards this, we analyzed the correlation between gut microbiota composition at the genus level and the percentage of lymphocyte subsets in MDS patients. The results showed that the abundance of *Phascolarctobacterium* was negatively correlated with the percentage of CD4^+^ T cells and the CD4^+^/CD8^+^ ratio in the MDS group. Meanwhile, the abundance of *Bacteroides* was negatively correlated with the CD4^+^/CD8^+^ ratio in all three MDS groups (total, HR, and LR MDS). In previous studies, the percentage of CD4^+^ T cells and CD4^+^/CD8^+^ ratios were reduced in MDS patients [32]. Therefore, we inferred that the decrease in the CD4^+^/CD8^+^ ratio might be induced due to the increased abundance of *Bacteroides* and that *Phascolarctobacterium* might regulate the percentage of CD4^+^ T cells thereby affecting the CD4^+^/CD8^+^ ratio in MDS. The abundance of *Parabacteroides* and *Enterobacter* was also negatively correlated with the CD4^+^/CD8^+^ ratio in the HR-MDS and LR-MDS groups, respectively. In the future, we wish to investigate the mechanisms by which altered gut microbiota influence CD4^+^ T cells and their subsets (such as Tregs and naïve and memory CD4^+^ T cells).

In the HR-MDS group, the abundance of *Haemophilus* and *Enterococcus* was negatively correlated with the percentage of CD8^+^ T cells and NK cells, respectively. The increased abundance of *Haemophilus* and *Enterococcus*, as opportunistic pathogenic microorganisms, might suggest potential infections and damage to cellular immunity by reducing the percentage of CD8^+^ T cells in HR-MDS. In lung cancer studies, *Veillonella* was enriched in cancer tissue samples that could activate the PI3K pathway *in vitro*, which is relevant for cancer pathogenesis of bronchial epithelial cell lines [33]. In HR-MDS, we found that the abundance of *Veillonella* increased and was negatively correlated with the percentage of CD8^+^ T cells. We inferred that *Veillonella* might participate in MDS progression by inhibiting CTLs in the microenvironment. In the future, we wish to investigate the effect of *Veillonella* on the signaling pathways involved in cancerization in MDS. These results indicate that immune effector cell impairment in HR-MDS might be related to gut dysbacteriosis that might promote immune evasion of malignant clones.

In the LR-MDS group, the percentage of total T cells positively correlated with the abundance of *Faecalibacterium*. In Crohn's disease patients, *Faecalibacterium* exerts anti-inflammatory effects by stimulating blood mononuclear cells [34]. However, the mechanisms underlying their effect on T cells in MDS are unclear. In addition, the percentage of CD8^+^ T cells was positively correlated with the abundance of both *Haemophilus* and *Enterobacter* and negatively correlated with that of *Streptococcus*. The presence of opportunistic pathogens, such as *Streptococcus* and *Haemophilus*, also suggests potential infections in LR-MDS. In contrast to HR-MDS, the increased abundance of *Haemophilus* was positively correlated with the percentage of CD8^+^ T cells in LR-MDS. *Haemophilus* has been shown to activate CD8^+^ T cells in children with otitis media [35]. This indicates that the cellular immune status of LR-MDS is different from that of HR-MDS. The upregulation of CD8^+^ T cells might damage hematopoietic cells, leading to cytopenia in LR-MDS. The function of *Enterobacter* in LR-MDS is unclear. In patients with Behçet's disease, the abundance of *Megamonas hypermegale* decreased and was associated with T cell aberration by metabolite alteration [36]. In LR-MDS, the decreased abundance of *Megamonas* might induce a decrease in the percentage of NK cells, the mechanisms of which require further study. These results indicate that the gut microbiome may have a regulatory effect on immune cells in patients with MDS.

Plasma metabolomics is also known to contribute to the development of hematological diseases. A distinct glucose metabolism signature is reported to be a novel prognostic marker and a potential therapeutic target in AML [37]. It generated a prognostic risk score with six metabolite markers suggesting enhanced glycolysis and the tricarboxylic acid cycle, further contributing to decreased sensitivity to cytarabine. In this study, we found that MDS patients had increased plasma concentrations of hypoxanthine and PYG and decreased concentrations of 3a,7a-dihydroxy-5b-cholestan, thereby indicating that these metabolites may be the characteristic metabolites of MDS. First, we identified increased levels of PYG in MDS. In recent studies, glutamine acyl peptide cyclotransferase-like protein (QPCTL) has been identified as the major component of the CD47-SIRP*α* pathway. Biochemical analysis showed that QPCTL is essential for the formation of PYG on CD47 at the SIRP*α* binding site [38]. We have previously reported that the abnormal hematopoietic stem cells of high-risk MDS patients overexpressed CD47 that could cause the malignant clones to avoid phagocytosis [39]. The increase in PYG may be related to increased CD47 expression in the malignant clones in MDS patients. The hypoxanthine level was also increased in patients with MDS. Hypoxanthine is involved in uric acid metabolism. Some studies have shown that the hypoxanthine level is positively correlated with the abundance of Firmicutes [40]. In our study, the abundance of Firmicutes was slightly increased in MDS patients, which might influence the level of hypoxanthine. However, the function of 3a,7a-dihydroxy-5b-cholestan in MDS is unclear. We also analyzed the correlation between plasma concentrations of these three metabolites and the percentage of immune subsets in patients with MDS. It showed some correlation trends, but these were not statistically significant, which may be because of limited sample size (Supplementary Figure 1).

Many studies have shown that modulation of the microbiome may affect responses to cancer therapy. Thus, it is possible to manipulate the gut microbiome to enhance therapeutic responses [41]. In hepatobiliary cancers, a higher abundance of *Ruminococcus callidus* and *Erysipelotrichaceae bacterium-GAM147* was found in patients with longer progression-free survival (PFS), while a higher abundance of *Veillonellaceae* was found in patients with shorter PFS and overall survival (OS). The gut microbiome is associated with the clinical response to anti-programmed cell death protein 1 (PD-1) therapy in patients with hepatobiliary cancers [42]. In metastatic melanoma, *Faecalibacterium* enrichment is associated with a good clinical response to ipilimumab (an immune checkpoint inhibitor targeting CTLA-4) [43]. This indicates that the gut microbiome might be a biomarker for predicting clinical response and can act as a novel therapeutic target in cancer immunotherapy. In allogeneic hematopoietic cell transplantation (allo-HCT), the gut microbiome is associated with clinical factors of acute graft-versus-host disease (GVHD) [44]. Fecal microbiota transplant (FMT) has become a potential therapeutic option for severe colitis associated with GVHD after allo-HCT. In a small case series, FMT alleviated the diarrhea observed in refractory GVHD patients. However, after FMT, the bacterial, fungal, and viral communities responded differently to GVHD [45–47]. Further, large sample sizes are required to ascertain the efficacy and safety of FMT in hematological diseases. In the future, it is necessary to determine the effect of FMT on MDS based on the understanding of the roles of the gut microbiome in MDS.

Gut dysbacteriosis may also be associated with metabolic abnormalities. In hepatobiliary cancers, the gut microbiome is related to the response to PD-1 inhibitor treatment. Functional annotation indicated that the taxa enriched in the clinical benefit response group were associated with energy metabolism, whereas those in the non-clinical benefit group were associated with amino acid metabolism, which may modulate the clinical response to immunotherapy [42]. This indicates that the gut microbiota might regulate host immunity by influencing the metabolism in cancer. Studies on the correlation between the gut microbiome and plasma metabolomics in MDS are ongoing.

In conclusion, the gut microbiome and plasma metabolomics were altered and the abundance of bacterial genera correlated with the percentage of immunocyte subsets in patients with MDS. This might be a potential molecular mechanism that triggers immune imbalance in MDS. Furthermore, it may provide new biomarkers and therapeutic targets for MDS.

## Figures and Tables

**Table 1:** Changed metabolites in the MDS group compared to the healthy control group.

Description	Molecular formula	$\frac{m}{z}$	Ion mode	Matching degree	Fold change
Hypoxanthine	C_5_H_4_N_4_O	137.05	ESI^+^	90.4	2.53
Pyroglutamic acid	C_5_H_7_NO_3_	128.04	ESI^−^	83.6	1.62
3a,7a-Dihydroxy-5b-cholestan	C_27_H_48_O_2_	405.37	ESI^+^	54.7	0.70

## Data Availability

The sequencing data used to support the findings of this study are available from the corresponding author upon request.

## Supplementary Materials

Supplementary Materials Supplementary Table 1: characteristics of the study subjects for gut microbiome analysis. Supplementary Table 2: characteristics of the study subjects for plasma metabolomic analysis. Supplementary Table 3: results of the richness and diversity analysis of the gut microbiome. Supplementary Figure 1: the correlation between plasma concentrations of three differentially identified metabolites and the percentage of immune subsets in MDS patients.
