# Supplementary material for: Gut Microbiome and Plasma Metabolomic Analysis in Patients with Myelodysplastic Syndrome
Source: Oxid Med Cell Longev. 2022 May 9;2022:1482811. doi: 10.1155/2022/1482811 (PMC9110251; doi:10.1155/2022/1482811)

**Supplementary Table**

Supplementary Table1. Characteristics of the study subjects for gut microbiome analysis.

|  | **MDS** | **Controls** |
| --- | --- | --- |
| Number | 15 | 14 |
| Gender(male/female) | 6/9 | 3/11 |
| Median age in years(range) | 65(18-69) | 43.5(26-63) |
| Subtype |  |  |
| SLD  RS | 1  4 |  |
| RCMD | 3 |  |
| EB-1 | 2 |  |
| EB-2 | 4 |  |
| 5q- | 1 |  |

Supplementary Table2. Characteristics of the study subjects for plasma metabolomics analysis.

|  | **MDS** | **Controls** |
| --- | --- | --- |
| Number | 10 | 10 |
| Gender(male/female) | 5/5 | 4/6 |
| Median age in years(range) | 59(26-71) | 30(25-38) |
| Subtype |  |  |
| SLD | 1 |  |
| RS | 2 |  |
| RCMD | 3 |  |
| EB-1 | 2 |  |
| EB-2 | 2 |  |

Supplementary Table 3. Results of the richness and diversity analysis of the gut microbiome

| Alpha  diversity index | MDS(n=) | Control(n=14) | p-vaule |
| --- | --- | --- | --- |
| Sobs | 155.66667 | 143.21429 | 0.52977 |
| Chao | 175.99387 | 163.85759 | 0.80945 |
| Ace | 178.37586 | 168.27365 | 0.83967 |
| Shannon | 2.70298 | 2.57708 | 0.18377 |
| Simpson | 0.19077 | 0.18683 | 0.23579 |
| Coverage | 0.99932 | 0.99934 | 0.95995 |

**Supplementary Figure Legends**

Supplementary Figure 1.The correlation between plasma concentrations of three differentially identified metabolites and the percentage of immune subsets in MDS patients.

**Supplementary Figures**

Supplementary Figure 1


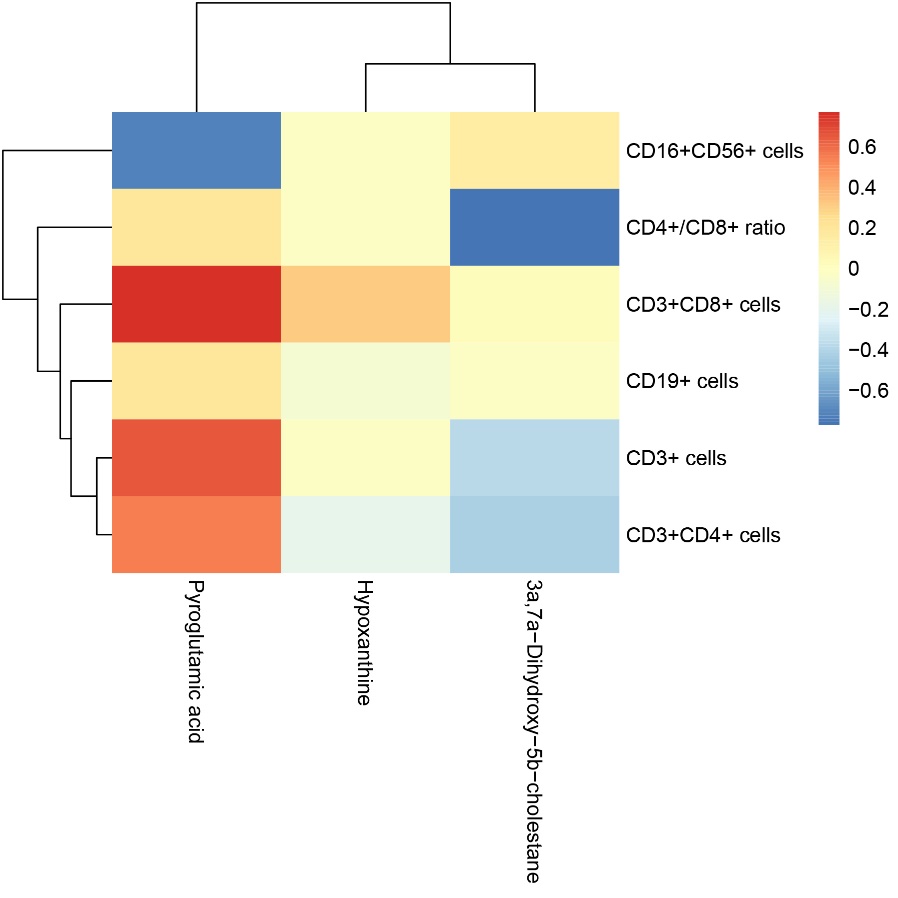

Supplement: Supplementary Materials — Supplementary Table 1: characteristics of the study subjects for gut microbiome analysis. Supplementary Table 2: characteristics of the study subjects for plasma metabolomic analysis. Supplementary Table 3: results of the richness and diversity analysis of the gut microbiome. Supplementary Figure 1: the correlation between plasma concentrations of three differentially identified metabolites and the percentage of immune subsets in MDS patients. [file 1482811.f1.docx]
